# Complete Genome Sequence of Melissococcus plutonius DAT561, a Strain That Shows an Unusual Growth Profile, Obtained by PacBio Sequencing

**DOI:** 10.1128/genomeA.00431-18

**Published:** 2018-06-07

**Authors:** Kayo Okumura, Daisuke Takamatsu, Masatoshi Okura

**Affiliations:** a Department of Veterinary Medicine, Obihiro University of Agriculture and Veterinary Medicine, Obihiro, Hokkaido, Japan; b Division of Bacterial and Parasitic Disease, National Institute of Animal Health, National Agriculture and Food Research Organization, Tsukuba, Ibaraki, Japan; c The United Graduate School of Veterinary Sciences, Gifu University, Gifu, Japan

## Abstract

Melissococcus plutonius is the causative agent of European foulbrood, and its isolates were believed to be remarkably genetically homogeneous. However, recent epidemiological and pathogenic studies have shown this pathogen to be more heterogeneous than expected. Herein, we present the whole-genome sequence of M. plutonius DAT561, a representative atypical strain.

## GENOME ANNOUNCEMENT

Melissococcus plutonius infects honeybee larvae and causes European foulbrood (EFB) (1). Because is it highly contagious and difficult to eradicate, this disease has been listed by the OIE (the World Organisation for Animal Health) as a notifiable bacterial honeybee disease, together with American foulbrood (http://www.oie.int/animal-health-in-the-world/oie-listed-diseases-2018/). A study comparing proteins and DNA fragment profiles demonstrated M. plutonius to be remarkably genetically homogeneous (2). However, M. plutonius-like organisms with different physiological characteristics are often isolated from diseased larvae showing typical clinical signs of EFB in Japan (3). As a striking example, some M. plutonius strains/isolates do not require high-potassium conditions for their normal growth, even though potassium phosphate has long been thought essential to the culture of this bacterium on media. In addition, one atypical M. plutonius strain was shown to be much more virulent toward honeybee larvae than are typical M. plutonius strains (4). To elucidate the genetic background of atypical M. plutonius strains, we sequenced the complete genome of M. plutonius DAT561, a representative atypical strain.

The sequencing was performed using a PacBio RS II platform (Pacific Biosciences, Menlo Park, CA, USA) in combination with the single-molecule real-time (SMRT) cell 8Pac version 3 and DNA polymerase binding kit P6 (Pacific Biosciences). We obtained a total of 90,627 reads covering a total of 752,156,879 bp. The mean subread length and *N*_50_ were 8,299 bp and 12,584 bp, respectively. The HGAP3 software (Pacific Biosciences) was used for *de novo* assembly, and sequences were assembled into two contigs that subsequent analysis showed to be chromosomal DNA and plasmid pMP1. Plasmid pMP19 was sequenced by Sanger sequencing with conventional primer walking. The pMP19 sequence was assembled with Sequencher 5.2 (Gene Codes Corp., Ann Arbor, MI, USA). Primary coding sequence (CDS) extraction and initial functional assignment were performed using the RASTtk automated annotation server (5). The PHASTER Web server was used to search phage DNA components in the DAT561 genome (6). The results were compared with genome sequences of M. plutonius ATCC 35311 and previous DAT561 sequences to verify annotation and were corrected manually using the *in silico* MolecularCloning software (In Silico Biology, Inc., Kanagawa, Japan).

The M. plutonius DAT561 genome is a single circular chromosome of 1,847,807 bp, with an average GC content of 31.5%. The chromosome contained a total of 1,531 CDSs, 18 pseudogenes, 55 tRNA genes for all amino acids, and four rRNA operons. In addition, the chromosome harbored four incomplete prophages. The genome contained two plasmids, pMP1 and pMP19, comprising 200,057 and 19,967 bp, respectively, with average GC contents of 29.2% and 30.3%, respectively, and pMP19 was partially sequenced. pMP1 and pMP19 contained 162 and 28 CDSs, respectively, and three pseudogenes were found in the pMP1 plasmid.

### Accession number(s).

The whole-genome sequences of the chromosome and two plasmids of M. plutonius DAT561 were deposited in DDBJ under accession numbers AP018492 (chromosome), AP018493 (pMP1), and AP018494 (pMP19).
